# Recommendations for fertility preservation in patients with lymphoma, leukemia, and breast cancer

**DOI:** 10.1007/s10815-012-9786-y

**Published:** 2012-05-31

**Authors:** ISFP Practice Committee, S. Samuel Kim, Jacques Donnez, Pedro Barri, Antonio Pellicer, Pasquale Patrizio, Zev Rosenwaks, Peter Nagy, Tommaso Falcone, Claus Andersen, Outi Hovatta, Hamish Wallace, Dror Meirow, Debra Gook, Seok H Kim, Chii-Ruey Tzeng, Shuetu Suzuki, Bunpei Ishizuka, Marie-Madeleine Dolmans

**Affiliations:** 1 Department of OB/GYN, University of Kansas Medical Center, 3901 Rainbow Blvd., Kansas City, KS 66212 USA; 2 Department of OB/GYN, Universite Catholique de Louvain, Brussels, Belgium; 3 Department of OB/GYN, Institut Universitari Dexeus, Barcelona, Spain; 4 Department of OB/GYN, Instituto Universitario IVI, Valencia, Spain; 5 Department of OB/GYN, Yale University, New Haven, CT 06511 USA; 6 Department of OB/GYN, Weill Cornell Medical College, New York, NY 10021 USA; 7 Reproductive Biology Associates, Atlanta, GA 30342 USA; 8 Department of OB/GYN, Cleveland Clinic, Cleveland, OH 44195 USA; 9 Department of OB/GYN, Copenhagen University Hospital, Copenhagen, Denmark; 10 Department of OB/GYN, Karolinska Institutet, Stockholm, Sweden; 11 Royal Hospital for Sick Children, Edinburgh, EH9 1LF UK; 12 Department of OB/GYN, Sheba Medical Center, Tel-Hashomer, Israel; 13 Reproductive Service, Royal Women’s Hospital, Parkville, Victoria Australia; 14 Department of OB/GYN, Seoul National University, Seoul, Korea; 15 Department of OB/GYN, Taipei Medical University Hospital, Taipei, Korea; 16 Tokyo, Japan; 17 Department of OB/GYN, St. Marianna University School of Medicine, Kawasaki, Kanagawa Japan

**Keywords:** Fertility preservation, Cancer, Lymphoma, Leukemia, Breast cancer, Chemotherapy, Ovarian tissue cryopreservation, Oocyte cryopreservation, Primary ovarian insufficiency, Cancer survival, Gonadal failure, GnRH agonist, Fertility

## Abstract

Fertility issues should be addressed to all patients in reproductive age before cancer treatment. In men, cryopreservation of sperm should be offered to all cancer patients in reproductive age regardless of the risk of gonadal failure. In women, the recommendation of fertility preservation should be individualized based on multiple factors such as the urgency of treatment, the age of the patient, the marital status, the regimen and dosage of cancer treatment.

Fertility issues should be addressed to all patients in reproductive age before cancer treatment. In cases where the patient is a minor, it is recommended to obtain assent from a patient in addition to signed consent from the parents. In men, cryopreservation of sperm should be offered to all cancer patients in reproductive age regardless of the risk of gonadal failure. In women, the recommendation of fertility preservation should be individualized based on multiple factors such as the urgency of treatment, the age of the patient, the marital status, the regimen and dosage of cancer treatment. If the risk of gonadal failure is very low (such as ABVD), fertility preservation may not be required. On the other hand, the patient who undergoes hematopoietic stem cell transplant (HSCT) should strongly consider fertility preservation. In principle, embryo cryopreservation or oocyte cryopresevtion is recommended as a fertility preservation option, if there is enough time for ovarian stimulation before initiation of cancer therapy. If cancer treatment cannot be delayed or ovarian stimulation is contraindicated, ovarian tissue cryopreservation should be the first option of fertility preservation.

## Incidence and survival (cited from http://seer.cancer.gov)

1. Lymphoma

Hodgkin lymphoma (HL): It is estimated that 4,010 women are newly diagnosed with HL in 2011. Among those, approximately 1,760 are under age 34. Approximately, 12.3 % were under age 20, 32 % between 20–34, and 15.8 % between 35–44. (2004–2008). The overall 5-year relative survival for 2001–2007 was 84 %. Currently, 5-year relative survival for women under age 49 is 90-95 %.

Non Hodgkin lymphoma (NHL): It is estimated that 30,300 women are diagnosed with NHL in 2011. Among those, approximately 1,670 are under age 34. Approximately 1.7 % were diagnosed under age 20, 3.8 % between 20–34, and 6.8 % between 35 and 44. The overall 5-year relative survival for 2001–2007 was 67 %. Currently, 5-year relative survival for women under age 49 is 80-85 %.

2) Leukemia

Acute lymphocytic leukemia (ALL): It is estimated that 2,410 women are newly diagnosed with ALL in 2011. Among those, approximately 1,750 are under age 34. Approximately, 60.3 % were under age 20, 10.3 % between 20–34, and 5.9 % between 35–44. (2004–2008). The overall 5-year relative survival for 2001–2007 was 64 %.

Chronic lymphocytic leukemia (CLL): The incidence is about 6,000/year, but it is very rare disease in women with age under 34 (0.3 %). The overall 5-year relative survival for 2001–2007 was 78 %.

Acute myeloid leukemia (AML): It is estimated that 6,120 women are diagnosed with AML in 2011. Among those, approximately 810 are under age 34. Approximately, 6.1 % were under age 20, 6.6 % between 20–34, and 6.6 % between 35–44. (2004–2008). The overall 5-year relative survival for 2001–2007 was 23 %.

Chronic myeloid leukemia (CML): It is estimated that 2,150 women are newly diagnosed with HL in 2011. Among those, approximately 220 are under age 34. Approximately, 2.6 % were under age 20, 7.7 % between 20–34, and 9.9 % between 35–44. (2004–2008). The overall 5-year relative survival for 2001–2007 was 57 %.

3) Breast Cancer

It is estimated that 230,480 women are newly diagnosed with breast cancer in 2011. Among those, approximately 11,000 are under age 40. From 2004–2008, 0 % were diagnosed under age 20, 1.9 % between 20–34, and 10.2 % between 35–44. The overall 5-year relative survival is approximately 90 %. In US there are approximately 2.6 million women alive with a history of breast cancer.

## Treatment and its effects on gonadal function

1. Lymphoma

Chemotherapy induced gonadal dysfunction depends on the age at first treatment and the treatment protocols. The risk of gonadal dysfunction after cancer treatment is low in pediatric population. NHL patients treated with chemotherapy (except HSCT) have very low risk of developing primary ovarian insufficiency (POI). Here are the common treatment regimens for HL and NHL.

### HL

*ABVD* (adriamycin, bleomycin, vinblastine, dacarbazine): POI rates are less than 10 % in the reproductive age; *BEACOPP* (bleomycin, etoposide, adriamycin, cyclophosphamide, oncovin, procarbazine, predisone): POI rates are around 50 % in those under 30 years; *MOPP* (chlormethamine, oncovine: procarbazine, prednisone): POI rates are 20- 50 % in women in reproductive age; *HSCT*: POI rates are 70-100 %, post-treatment parenthood rates are as low as 3-8 % [1].

### NHL

*CHOP* (cyclophosphamide, doxorubicin, vincristine, predisone): POI rates are around 5 %, pregnancy rates after treatment are 50 % [2]; *Hyper-CVAD* (cyclophosphamide, vincristine, doxorubicin, dexamethasone, cytarabine, methotrexate): POI rates are 14 %, pregnancy rates after treatment are 43 % [3]; *HSCT*: POI rates are 70-100 % [1].

2) Leukemia

The risk of infertility in patients with ALL or AML (unless treated with HSCT) is very low as contemporary treatment protocols entails lower doses of alkylating agents or are devoid of alkylating agents. CML can be treated with tyrosin kinase inhibitors such as imatinib or rituximab. Tyrosin kinase inhibitors may not impair fertility in humans, but there is insufficient data on these medications on reproductive potential. Of note, tyrosin kinases are important for follicle growth and development.

3) Breast cancer

Adjuvant chemotherapy after surgery or neo-adjuvant chemotherapy before surgery has an important role in breast cancer treatment, as it is likely to reduce the risk of recurrence and death from breast cancer by a relative factor of 50 % or more [4]. The primary factors affecting chemotherapy induced gonadotoxicity are age of the patient, and dose and number of cycles of the alkylating agent received. For example, the risk of amenorrhea after six cycle of *CMF* (cyclophosphamide, methotrexate, fluorouracil) or four cycyles of *AC* (anthracycline, cyclophosphamide) is 33 %, on the other hand, 50-65 % of patients will experience amenorrhea after treatment with six cycles of *FEC* (fluorouracil, epirubicin, cyclophosphamide), or *FAC* (fluorouracil, doxorubicin, cyclophosphamide), or four cycles of *AC* followed by four of *docetaxel* [4].

## Recommendations for fertility preservation

All patients who desire to preserve fertility should be counseled and informed about currently available fertility preservation options by fertility specialists. Recommendations should be individualized and should not violate the ethical principles. In general, fertility preservation before cancer treatment is strongly recommended if the chance of losing fertility is over 30 % with cancer therapy. In pediatric patients, the risk of gonadal failure with chemotherapy is very low in the absence of HSCT.

1. Lymphoma

Post-pubertal male: Cryopreservation of spermatozoa. GnRHa co-treatment is not recommended in male.

Pre-pubertal male: No good option. Cryopreservation of testicular tissue may be available in some centers as a strictly experimental procedure.

Post-pubertal female: Cryopreservation of embryos or cryopreservation of oocytes is recommended if cancer treatment can be delayed. However, immediate treatment is required in most of lymphoma patients and thus cryopreservation of ovarian tissue should be considered as a fertility preservation option. Alternatively, immature oocyte retrieval followed by IVM and cryopreservation of oocytes or embryos can be considered. The protective effect of GnRHa is questionable and controversial. However, GnRHa co-treatment can be considered for female patients undergoing chemotherapy (not for HSCT) if there is no other option.

Pre-pubertal female: Ovarian tissue cryopreservation, if the risk of ovarian failure after cancer treatment is high enough to justify the procedure.

2) Leukemia

Post-pubertal male: cryopreservation of spermatozoa.

Pre-pubertal male: no currently available option.

Post-pubertal female: No ideal option to date. However, cryopreservation of ovarian tissue should be considered before HSCT. Any harvested tissue from leukemia patients should not be used for auto-transplantation because of high risk of cancer cell reintroduction. Alternatively, immature oocyte retrieval followed by IVM and cryopreservation of oocytes or embryos can be considered.

Pre-pubertal female: Ovarian tissue cryopreservation before HSCT. Any harvested tissue from leukemia patients should not be used for auto-transplantation because of high risk of cancer cell reintroduction. In the absence of HSCT, fertility preservation before chemotherapy is not necessary.

3) Breast cancer

It is recommended that fertility preservation consultation is arranged at the time of initial diagnosis. In many cases, young breast cancer patients require adjuvant chemotherapy after surgery (mastectomy or lumpectomy). The best time for fertility preservation is after surgery and before adjuvant therapy. Cryopreservation of embryos or cryopreservation of oocytes is recommended as a fertility preservation option before chemotherapy. As cryopreservation of embryos or oocytes requires controlled ovarian stimulation (COS), the risk of increased peak estradiol levels with COS in breast cancer patients (especially with ER + tumor) should be discussed before the procedure. The COS strategy using tamoxifen or letrozole in conjunction with gonadotropin may be safer for women with ER + tumor. For women who require urgent cancer treatment such as neo-adjuvant chemotherapy, cryopreservation of ovarian tissue should be considered. Alternatively, immature oocyte retrieval followed by IVM and cryopreservation of oocytes or embryos can be considered.

## (Addendum)

Criteria for ovarian tissue banking (by S. Samuel Kim)

1. Age: under 37 years (may be individualized based on the status of ovarian reserve)
2. Ovarian function: premenopausal by FSH, antral follicle count (AFC) or AMH
3. Communication with oncologists: cancer treatment plan, prognosis
4. When embryo freezing or oocyte freezing is not indicated: delaying cancer treatment is not acceptable, hormonal stimulation is not permitted, ART is not allowed.
5. Prepubertal girls who do not have any other options
6. High risk for POF (when significant loss of ovarian follicles is anticipated with cancer therapy)
7. Informed consent from adult patients
8. Informed consent from parents/guardians as well as informed assent from minors, if the patient is less than 18 years
9. Physically and mentally healthy enough for surgery
10. Desires to have a child in the future (preferably before the age 50)
11. Thorough patient counseling: currently available fertility preservation options including embryo and oocyte cryopreservation, how to use cryobanked ovarian tissue for fertility restoration
12. Should understand experimental nature and potential risks of cancer cell transmission.
